# The Tilburg double blind randomised controlled trial comparing inguinal hernia repair according to Lichtenstein and the transinguinal preperitoneal technique

**DOI:** 10.1186/1745-6215-10-89

**Published:** 2009-09-25

**Authors:** Giel G Koning, Hans JP de Schipper, Henk JM Oostvogel, Michiel HJ Verhofstad, Pieter G Gerritsen, Kees CJHM van Laarhoven, Patrick WHE Vriens

**Affiliations:** 1 Department of Surgery, St Elisabeth Hospital, LC Tilburg, The Netherlands

## Abstract

**Background:**

Anterior open treatment of the inguinal hernia with a tension free mesh has reduced the incidence of recurrence and direct postoperative pain. The Lichtenstein procedure rules nowadays as reference technique for hernia treatment. Not recurrences but chronic pain is the main postoperative complication in inguinal hernia repair after Lichtenstein's technique. Preliminary experiences with a soft mesh placed in the preperitoneal space showed good results and less chronic pain.

**Methods:**

The TULIP is a double-blind randomised controlled trial in which 300 patients will be randomly allocated to anterior inguinal hernia repair according to Lichtenstein or the transinguinal preperitoneal technique with soft mesh. All unilateral primary inguinal hernia patients eligible for operation who meet inclusion criteria will be invited to participate in this trial. The primary endpoint will be direct postoperative- and chronic pain. Secondary endpoints are operation time, postoperative complications, hospital stay, costs, return to daily activities (e.g. work) and recurrence. Both groups will be evaluated.

Success rate of hernia repair and complications will be measured as safeguard for quality.

To demonstrate that inguinal hernia repair according to the transinguinal preperitoneal (TIPP) technique reduces postoperative pain to <10%, with α = 0,05 and power 80%, a total sample size of 300 patients was calculated.

**Discussion:**

The TULIP trial is aimed to show a reduction in postoperative chronic pain after anterior hernia repair according to the transinguinal preperitoneal (TIPP) technique, compared to Lichtenstein.

In our hypothesis the TIPP technique reduces chronic pain compared to Lichtenstein.

**Trial registration:**

ISRCTN 93798494

## Background

Inguinal hernia is a common surgical problem. In the Netherlands more than 26.000 unilateral inguinal hernia repairs are performed each year [1]. Tension free mesh repair has reduced the incidence of recurrence and direct post operative pain. The incidence of recurrences are 2-5% [2]. However, chronic pain after inguinal hernia repair is an underestimated problem [3].

The exact incidence of chronic pain is unknown. Well conducted, large and unselected epidemiological studies suggest that about 20% of patients are affected with chronic pain [4-7]. A randomised controlled trial comparing Shouldice, Lichtenstein and endoscopic preperitoneal repair showed that 31% of patients had some form of chronic pain after Lichtenstein repair [8]. Patients are classified as having chronic pain if postoperative pain lasts for more than three months [9]. Chronic pain may vary from subtle discomfort to disabling pain. In general, three chronic groin pain syndromes have been defined: somatic, neuropathic, and visceral pain [10]. Somatic pain is localized to the pubic tubercle and is a result of periosteal damage during stapling of prosthetic mesh or incorporation of the peri-osteum into the most medial stitch of an open anterior repair. Neuropathic pain usually develops in the sensory distribution of the injured nerve and can present days to weeks after the repair. Chronic neuralgia results from nerve trauma secondary to partial or complete division, stretching, contusion, crushing, electrical damage, suture compression, and adjacent inflammation from mesh or suture material [11,12]. The most commonly offended nerves after open inguinal hernia repair include the ilioinguinal-, iliohypogastric-, and genital branch of the genitofemoral nerve [13]. Visceral pain usually presents as chronic orchalgia, or pain during ejaculation, and can be a result of stricture of the spermatic duct or damage to the somatic sacral or sympathetic nerves [14].

To assess the objectivity of pain the visual analogue scale (VAS score) is frequently used nowadays [15]. VAS has been a proven instrument to score postoperative pain in inguinal surgery. Chronic pain has significant effects on all daily activities including walking, work, sleep, relationships with other people, mood and general enjoyment of life [16]. So much effort has been put in strategies to reduce chronic pain.

Endoscopic hernia repair has been postulated to result in less chronic pain due to the preperitoneal placed position of the mesh.

Several studies have been performed to investigate if endoscopic repair resulted in less chronic pain. A large mesh is placed in preperitoneal position to cover the myopectineal orifice after reduction of the hernia sac. Liem et al. concluded a lower incidence of pain after endoscopic hernia repair compared to open non-mesh repair [17]. Grant et al. however found in a randomised trial after 5 years that the incidence of chronic pain was still 27% at one year compared with 36% chronic pain in the Lichtenstein group. Groin numbness was significantly reduced (18% vs 40%) [18]. Specialized hernia centres reported excellent results after endoscopic repair. Wright failed to prove reduced pain after endoscopic approach (13% vs. 10%) in a randomised controlled trial with 5 years follow up. This study showed that testicular pain was more frequent after endoscopic repair and groin pain more common after open repair [19]. A review of randomised controlled trials comparing endoscopic with open mesh hernia repair showed that the endoscopic approach was associated with less persisting pain [2].

The drawback of endoscopic hernia repair over the open approach is the added costs, particularly when disposable instruments are used [20]. The other disadvantage of laparoscopy compared with open hernia repair is that general anaesthesia is necessary. Finally, the endoscopic learning curve is long [21]. The principal reasons for the long learning curve are the surgeon's lack of familiarity with the preperitoneal anatomy and the time it takes to develop the skills to operate in a confined space. Complications of endoscopic hernia repair are scarce but might be severe.

One study prospectively investigated the incidence of pain after inguinal hernia correction under local anaesthesia. Some form of pain was reported by 19% of patients and severe pain by 6% of patients after one year [22].

Other meshes have been developed and tested to reduce the chronic pain after inguinal hernia repair. A randomised clinical trial comparing the Prolene Hernia System, mesh plug repair and Lichtenstein method for open inguinal hernia repair showed that 39,7% of patients had some form of pain after three months. No difference could be detected between type of mesh used [23].

### Rationale for the soft mesh

No recent systematic review or meta-analysis regarding postoperative pain after inguinal hernia repair has been performed.

Endoscopic preperitoneal approach in order to reduce pain is expensive and has several other disadvantages, but a cost-effectiveness meta analysis has to be performed yet.

Since pain is often related to neuralgia and recurrences occur at the myopectineal orifice an alternative mesh was developed to be placed in the preperitoneal space, but with anterior approach. Preliminary experience with a preperitoneal placed mesh of 8-10 cm long and 6-7 cm wide in 116 patients showed a recurrence rate of 0,7% with a median follow up of 63 months [24]. After adding a memory ring to the mesh to allow easy placement in the preperitoneal space, preliminary results in 126 patients with a median follow up of 24 months show a recurrence rate of 1,6% and reported pain in 5,6% of patients [25]. A prospective study on feasibility and postoperative outcome of this memory ring-equipped-mesh showed that this method could be achieved in all 200 patients and with a low risk of postoperative complications (7% benign, of which 10 patients complained of mild pain postoperatively) [26].

The transinguinal preperitoneal (TIPP) technique with soft mesh combines the anterior approach according to Lichtenstein with the preperitoneal position of the mesh as known from the endoscopic total extra peritoneal technique (TEP).

This paper describes the rationale of this product and the design of the study.

## Methods

### Study objectives

Two techniques, Lichtenstein and TIPP, for inguinal hernia patients will be compared in a prospective randomised double blind clinical trial. Patients will be included at the outpatient departments of both hospitals by surgeons and supervised residents. Dedicated hernia surgeons will always supervise and/or perform the operations.

Objective success: the percentage of operations with successful inguinal hernia reduction and direct postoperative - and chronic pain incidence lower than 25%.

### Primary endpoint

The primary endpoint is the incidence of direct postoperative pain and chronic pain after inguinal hernia repair according to Lichtenstein or TIPP using the visual analogue scale (VAS).

### Secondary endpoints

The secondary endpoints are operation time, hospital stay, complications (e.g. infection), cost-efficiency analyses, time to return to daily activities/work and recurrence after Lichtenstein or TIPP procedures.

### Design

TULIP is a double-blind, randomised controlled trial. Randomisation will be performed by pulling a sealed double blind envelope in the trial centre after contact by telephone prior to incision. Obviously, the surgeons know which technique they are performing during operation. The investigator assessing the outcome on the outpatient department is not informed. Operation reports are blinded and no (digital) access is permitted or possible for the person assessing outcomes. The patient does not know which procedure has been performed because it is written down on paper and not mentioned in theatre.

### Patients

**Figure 1:**
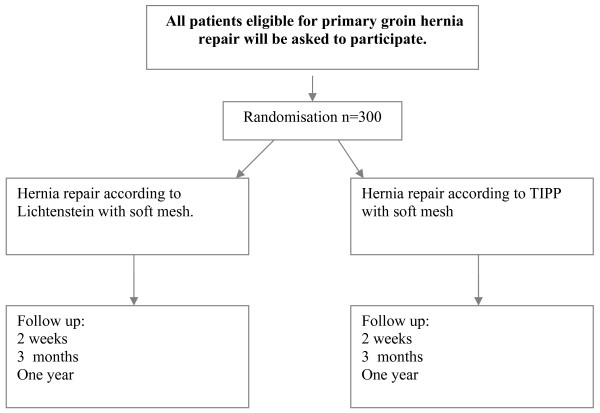


A total of 300 patients, with a unilateral primary groin hernia, visiting the outpatient clinics at the St. Elisabeth Hospital or TweeSteden Hospital in Tilburg will be randomised (figure 1).

Analyses of inguinal hernia patients in the past showed a number of approximately 600-700 per year in both hospitals, equally divided. This number is the sum of both hospitals and includes recurrences, children/elderly, incarcerated and bilateral inguinal hernia's. Apart from a positive informed consent, a lower amount can/will be included. Data will be collected by VAS-diary and SF36-list (Health Status/Quality of Life). Forms will be filled in by the patients at 14 days, 3 months and one year after surgery during follow up (figure 1).

### Inclusion criteria

• Primary unilateral groin hernia

• Age > 18, < 80 years

• ASA classification 1-3

• Signed informed consent letter

### Exclusion criteria

• Recurrent hernia

• Age <18 or >80 years

• Scrotal hernia(s)

• ASA classification >4

• Acute incarcerated inguinal hernia(s)

• Psychiatric disease or other reason making follow up or questionnaires unreliable

• Previous preperitoneal surgery (e.g. radical prostatectomy)

### Ethics, informed consent

This study is conducted in accordance with the principles of the Declaration of Helsinki and "Good Clinical Practice Guidelines". The independent ethics committee of both participating hospitals approved the final protocol. Oral and written informed consent in form is obtained from the patient before inclusion in the trial.

The TULIP Trial is registered at: .

### Surgical Techniques

All patients will be operated via anterior approach with a skin incision two centimetres above the Poupart ligament. In half of the study population the groin hernia will be corrected according to Lichtenstein, as described by Amid et al. [27]. This is the reference treatment advised by the Dutch Society of Surgeons [28]. The Lichtenstein technique will be attempted to present-day insights; a soft mesh will be used instead of the polypropylene mesh [29].

The other 150 inguinal hernia patients will be operated by the transinguinal preperitoneal (TIPP) technique with Polysoft^® ^mesh as described by Pélissier et al. [26]. In this technique an inguinal incision 8-10 cm long is made, the external oblique aponeurosis is divided and the cord lifted on a tape. The cremaster muscle is divided around the internal orifice, but not striped, and the sac is dissected. The technique of placement of the Polysoft^® ^mesh into the preperitoneal space adapts anatomically to the type of hernia. Type of hernia will be assessed using the European Hernia Society groin hernia classification [30]. This classification is simple and easy to remember. The size of the hernia orifice is registered as 1 (≤ 1 finger), 2 (1-2 fingers) or 3 (≥ 3 fingers) accompanied with L (lateral), M (medial) or F (femoral). All the hernia's will be primary (P) classified according to the inclusion criteria so recurrent (R) will not be assessed in our population. Example; a lateral inguinal hernia with an orifice of 2 fingers and a primary origin will result into L2P.

In indirect hernias high dissection of the sac is performed and the sac is thus reduced in the pre peritoneal space (PPS) through the internal ring. Blunt dissection is carried out in the PPS, through the internal orifice and is then extended deep to epigastric vessels and transverse fascia, in the direction of the pubic spine, beyond its level. The patch is introduced in the PPS via the internal orifice. In regional or local anaesthesia asking the patient to strain allows correct anatomical spreading of the mesh, which is applied to the deep aspect of the fascia. The assessment is done by asking the patient to strain and to cough. External oblique aponeurosis was repaired superficial to the cord to restore the normal anatomy.

In direct hernias, after division of the cremaster so as to check the internal orifice for an indirect sac, the transverse fascia is divided circularly around the hernia bulge and the sac is reduced. Blunt dissection is carried out in the PPS, medially in the direction of the pubic spine and laterally behind the epigastric vessels in direction of the iliac spine. The patch is introduced through the transverse fascia opening and spread in the PPS so as to cover all the weak inguinal area. When an indirect sac, even if it is small, is associated to the direct one, both sacs are dissected and reduced.

### Escape medication

A standardized general anesthesia/spinal anesthesia protocol will be used in both groups in combination with a standardized post operation regimen, based on VAS scores for pain and nausea. These regimes are based on current and acceptable practice and the standardization serves to avoid unnecessary bias.

The choice of anesthesia technique will be left, in principle, to the preference of the patient. All patients will be seen, pre-operatively at the pre-operative screening outpatient clinic at one of the two locations. Standardized pre- and postoperative medication will be handed out to the patients to be used each day, during the first 5 days postoperatively, including any part of this period that the patient may already have been discharged. This includes, but is not limited to, paracetamol 1 gram four times daily and diclofenac 50 mg 3 times daily and as rescue medication zaldiar 50 mg 3 times daily when regular painkillers are not satisfying.

### Statistical analysis

The analysis will be performed on the basis of intention-to-treat principles.

It is anticipated that the use of TIPP technique with soft mesh at least will lead to a reduction in postoperative chronic pain from 25% to 10%. The sample size calculation is based on α = 0,05 and a power of 80%. This leads to a required sample size of 300 patients. Taking into account a 5% loss-to-follow up, a total of 2 × 158 patients will be randomized. There are three postoperative follow up visits at the outpatient department at two weeks, three months and one year for both groups (Lichtenstein and TIPP). The expected study end is December of 2010 (after 1,5 years inclusion period).

### Randomisation

The randomisation list was generated by using the website randomisation.com . According to this list a random allocation of Lichtenstein and TIPP method was performed. Randomisation will take place by pulling a sealed envelope after phone call to the trial centre prior to incision at the theatre.

Operation forms are blinded in the electronic patients files and not available for the independent outpatient clinic researcher. The incision of the skin and anterior approach will be the same for both techniques.

## Conclusion

The TULIP is a double blind randomised controlled trial that aims to show a reduction in direct postoperative- and chronic pain after anterior inguinal hernia repair with placement of a soft mesh either in the inguinal canal (Lichtenstein) or in the preperitoneal space (TIPP).

Hypothetically the TIPP technique reduces chronic pain drastically compared to Lichtenstein because of the placement of the soft mesh in the preperitoneal space.

## Competing interests

The authors declare that they have no competing interests. Objective analyses will be performed on the TULIP data. There will be no violating of this study protocol.

## Authors' contributions

GGK and PWHEV were involved in trial coordination, design protocol, writing manuscript.

HJPS and PWHEV co-write manuscript "surgical technique" and reference manager. HJMO and PGG made comments on rough version manuscript. HJMO, KCJHML and PWHEV made comments on protocol and manuscript. KCJHML co-supervised protocol, conceiving study design, methods. PWHEV was the supervisor of protocol, manuscript and outpatient department. All authors approved the study and this manuscript.

## References

[B1] (2005). Source.

[B2] McCormack K, Scott NW, Go PM, Ross S, Grant AM, the EU Hernia Trialists Collaboration (2003). Laparoscopic techniques *versus *open techniques for inguinal hernia repair. Cochrane Database Syst Rev.

[B3] Fränneby U, Sandblom G, Nordin P, Nyre'n O, Gunnarsson U (2006). Risk Factors for Long-term Pain After Hernia Surgery. Ann Surg.

[B4] Bay-Nielsen M, Perkins FM, Kehlet H (2001). Pain and functional impairment 1 year after inguinal herniorrhaphy: a nationwide questionnaire study. Ann Surg.

[B5] Poobalan AS, Bruce J, Smith WC, King PM, Krukowski ZH, Chambers WA (2001). Chronic pain and quality of life following open inguinal hernia repair. Br J Surg.

[B6] Hair A, Duffy K, Mclean J, Taylor S, Smith H, Walker A (2000). Groin hernia repair in Scotland. Br J Surg.

[B7] Bay-Nielsen M, Nilsson E, Nordin P, Kehlet H (2004). Chronic pain after open mesh and sutured repair of indirect hernia in young males. Br J Surg.

[B8] Köninger J, Redecke J, Butters M (2004). Chronic pain after hernia repair: a randomized trial comparing Shouldice, Lichtenstein and TAPP. Arch Surg.

[B9] International Association for the Study of Pain (1986). Classification of chronic pain. Descriptions of chronic pain syndromes and definitions of pain terms. Prepared by the International Association for the Study of Pain, Subcommittee on Taxonomy. Pain.

[B10] Poobalan AS, Bruce J, Smith WC, King PM, Krukowski ZH, Chambers WA (2003). A review of chronic pain after inguinal herniorrhaphy. Clin J Pain.

[B11] Lichtenstein IL, Shulman AG, Amid PK, Montllor MM (1988). Cause and prevention of postherniorrhaphy neuralgia: a proposed protocol for treatment. Am J Surg.

[B12] Heise CP, Starling JR (1998). Mesh inguinodynia: a new clinical syndrome after inguinal herniorrhaphy?. J Am Coll Surg.

[B13] Bower S, Moore BB, Weiss SM (1996). Neuralgia after inguinal hernia repair. Am Surg.

[B14] Rosen MJ, Novitsky YW, Cobb WS, Kercher KW, Heniford T (2006). Combined open and laparoscopic approach to chronic pain following open inguinal hernia repair. Hernia.

[B15] Loos MJ, Houterman S, Scheltinga MR, Roumen RM (2008). Evaluating postherniorrhaphy groin pain: Visual Analogue or Verbal Rating Scale?. Hernia.

[B16] Corney CA, Duffy K, Serpell MG, O'Dwyer PJ (2002). Outcome of patients with severe chronic pain following repair of groin hernia. Br J of Surg.

[B17] Liem MS, Graaf Y van der, van Steensel CJ (1997). Comparison of conventional anterior surgery and laparoscopic surgery for inguinalhernia repair. N Engl J Med.

[B18] Grant AM, Scott NW, O'Dwyer PJ, on behalf of the MRC Laparoscopic Groin Hernia Trial Group (2004). Five-year follow-up of a randomized trial to assess pain and numbness after laparoscopic or open repair of groin hernia. Br J of Surg.

[B19] Wright D, Paterson C, Scott N, Hair A, O'Dwyer PJ (2002). Five-Year Follow-Up of Patients Undergoing Laparoscopic or Open Groin Hernia Repair A Randomized Controlled Trial. Ann Of Surg.

[B20] MRC Laparoscopic Groin Hernia Trial Group (No Authors Listed) (1999). Laparoscopic versus open repair of groin hernia: A randomised comparison. Lancet.

[B21] Wright D, O'Dwyer PJ, Cuschieri A, MacFadyen BV, Jr (1998). The learning curve for laparoscopic hernia repair. Seminars in laparoscopic surgery.

[B22] Callesen T, Bech K, Kehlet H (1999). Prospective study of chronic pain after groin hernia repair. Br J Surg.

[B23] Nienhuijs SW, van Oort I, Keemers-Gels MEL, Strobbe JA, Rosman C (1999). Randomized clinical trial comparing the Prolene Hernia System, mesh plug repair and Lichtenstein method for open inguinal hernia repair. Br J Surg.

[B24] Pelissier EP, Blum D, Marre D, Damas JM (2001). Inguinal hernia: a patch covering only the myopectineal orifice is effective. Hernia.

[B25] Pelissier EP (2006). Inguinal hernia: preperitoneal placement of a memory-ring patch by anterior approach. Hernia.

[B26] Pelissier EP, Monek O, Blum D, Ngo Ph The Polysoft patch: prospective evaluation of feasibility, postoperative pain and recovery. Hernia.

[B27] Amid PK, Shulman AG, Lichtenstein IL (1996). Open 'tension-free' repair of inguinal hernias: the Lichtenstein technique. Eur J Surg.

[B28] Simons MP, de Lange D, Beets GL, van Geldere D, Heij HA, Go PM (2003). The 'Inguinal Hernia' guideline of the Association of Surgeons of the Netherlands. Ned Tijdschr Geneesk.

[B29] Koch A, Bringman S, Myrelid P, Smeds S, Kald A (2008). Randomized clinical trial of groin hernia repair with titanium-coated lightweight mesh compared with standard polypropylene mesh. Br J Surg.

[B30] Miserez M, Alexandre JH, Campanelli G, Corcione F, Cuccurullo D, Hidalgo PM, Hoeferlin A, Kingsnorth AN, Mandala V, Palot JP, Schumpelinck V, Simmermacher RK, Stoppa R, Flament JB (2007). The European Hernia Society groin hernia classification: simple and easy to remember. Hernia.

